# Barriers Experienced by First Nations Deaf People in the Justice System

**DOI:** 10.1093/jdsade/enae021

**Published:** 2024-06-02

**Authors:** Brent C Elder, Karen Soldatić, Michael A Schwartz, Jody Barney, Damien Howard, Patrick McGee

**Affiliations:** Department of Wellness and Inclusive Services in Education, Rowan University, 201 Mullica Hill Dr., Glassboro, New Jersey 08028, United States; Canada Excellence Research Chair in Health Equity and Community Wellbeing (CERC), Toronto Metropolitan University, Institute of Culture and Society, Western Sydney University (Institute Fellow), 288 Church Street Toronto ON Canada M5B 1M5, Australia; College of Law, Syracuse University, 950 Irving Ave, Syracuse, New York 13244, United States; The Winston Churchill Memorial Trust, GPO Box 1536, Canberra ACT 2601, Australia; Phoenix Consulting, PO Box 793 Nightcliff 0814, Australia; The Winston Churchill Memorial Trust, GPO Box 1536, Canberra ACT 2601, Australia

## Abstract

Anecdotal evidence strongly suggests that members of the First Nations Deaf community experience more barriers when engaging with the criminal justice system than those who are not deaf. Therefore, our purpose for writing this article is to highlight legal and policy issues related to First Nations Deaf people, including perspectives of professionals working with these communities, living in Australia who have difficulty in accessing supports within the criminal justice system. In this article, we present data from semi-structured qualitative interviews focused on four key themes: (a) indefinite detention and unfit to plead, (b) a need for an intersectional approach to justice, (c) applying the maximum extent of the law while minimizing social services–related resources, and (d) the need for language access and qualified sign language interpreters. Through this article and the related larger sustaining project, we seek to center the experiences and needs of First Nations Deaf communities to render supports for fair, just, and equitable access in the Australian criminal justice system to this historically marginalized group.

There is a growing body of scholarship, advocacy, and activism that examines the impact of longstanding settler colonial racialization on Indigenous incarceration in what is now known as the country called Australia. Indigenous incarceration in settler colonial Australia is one of the highest Indigenous population groups to be incarcerated across settler colonial contexts (Royal Commission Report on Disability, 2023). In Canada, another former British colony, Indigenous populations represent 32% of the prison population, despite being 5% of the total population (Department of Justice Canada, 2023; Penal Reform International, 2022). In New Zealand, also a former British colony, Indigenous populations are three times more likely to be incarcerated (Ara Poutama Aotearoa Department of Corrections, 2023). In the United States, the incarceration of Indigenous populations is four times more than white people (Prison Policy Initiative, 2023). Even though Indigenous Australians represent approximately only 3.8% of the Australian population (Australian Bureau of Statistics [ABS], 2021), at the time of writing, Indigenous Australians were disproportionately represented within the prison population at around 32% of the incarcerated population overall (ABS, 2022; Penal Reform International, 2022).

There is increasing evidence that strongly articulates that disability prevalence across Indigenous populations is one of the primary reasons for the overrepresentation of Indigenous incarceration across settler colonial contexts. Recent reporting within the Australian Royal Commission into the violence, abuse, neglect, and exploitation of persons with disabilities shows that Indigenous Australians with disability are overrepresented within the prison population (Disability Royal Commission, 2020). Some researchers and civil society groups suggest that First Nations people with intellectual disability are the most vulnerable to indefinite incarceration (Dowse et al., 2021; Shepherd et al., 2017). Public submissions by disability rights and civil society groups suggest “that 95% of First Nations people charged with criminal offenses who appear before courts have an intellectual disability, a cognitive disability, or a mental illness” (Disability Royal Commission, 2020, p. 3). There is a large number of those who are in this category, including youth, that have undiagnosed hearing-related disabilities, with those in the justice system mistaking nonverbal communication as a form of intellectual disability instead of deafness (Australian Institute of Health and Welfare, 2022; Quinn & Rance, 2009; Vanderpoll & Howard, 2012). Additionally, the prevalence of those labeled as hard-of-hearing among this cohort is often due to a lack of assessment of hearing health in various systems (e.g., education, health care) including in the prison system (Deafness Forum Australia, 2022). Further, many inmates refuse assessments due to the lack of culturally appropriate interventions (e.g., Aboriginal language speakers/signers in detention are provided with interpreters, consulting with Elders in Aboriginal communities on supporting detained members of their communities) (Barney, 2016b; North Australian Aboriginal Justice Agency [NAAJA], 2017). These factors, among others, contribute to First Nations people with a variety of disabilities facing a criminal justice system that is not equipped for equitable representation before the law, which ultimately is a system of *injustice* for this population (Brolan & Harley, 2018).

Long-term evidence clearly indicates that high rates of hearing-related disabilities for incarcerated Indigenous populations plays a crucial role in negative engagements with policing, experiences, and entanglements with the criminal justice system, and the high rates of Indigenous recidivism (Deafness Forum Australia, 2022; Disability Royal Commission, 2019; Howard & Barney, 2020; Northern Territory Reform, 2016; Royal Commission and Board of Inquiry, 2017). The lack of appropriate accessible communication from an early age, such as the development of appropriate sign language within remote Indigenous households, impacts First Nations Deaf^1^ Australians’ experiences and outcomes in relation to schooling, community inclusion, and employment. Indeed, the lack of Australian Government commitment to better supporting First Nations Deaf sign language communication may contribute to high rates of incarceration across the life course (O’Brien & Trudgett, 2020).

This lack of government investment in First Nations Deaf communication development, especially in rural and remote regions, where a high proportion of Indigenous Australians live in connection to their country lands and waterways, contravenes Australia’s commitments to the realization of Indigenous rights and disability rights at the international level and in domestic law. In 1992, Australia enacted the Disability Discrimination Act (DDA), a domestic law that prohibits discrimination against people with disabilities, and in 2008, the country ratified the United Nations (UN) Convention on the Rights of Persons with Disabilities (UNCRPD, 2006 and the Optional Protocol in 2009,^2^ committing the country to comply with international human rights law protecting people with disabilities. Australia’s CRPD Committee Report on Article 13 recognizes that effective access to justice for persons with disabilities is crucial in ensuring inclusion and equality (Committee Report on the Rights of Persons with Disabilities, 2012, p. 17). An issue we explore in this article is how well Australia is in compliance with the CRPD given the concerningly high rates of incarceration for First Nations Deaf people.

Further, in 2009, the Australian Government endorsed the United Nations Declaration on the Rights of Indigenous Peoples (UNDRIP) (2007), and has, since this time, continually purported to claim commitments to Indigenous rights in a range of international forums (see Australian Human Rights Commission [AHRC], 2021). Yet, despite these articulated commitments, until the recent election of the Federal Labor Party to National Parliament, no prior Federal Governments in power of any persuasion have made efforts for the full realization of the UNDRIP in domestic law, policy, and programs (AHRC, 2021). Even with the ardent mobilization of Indigenous Australians through a combination of activist street protests and formal advocacy and lobbying placing pressure on previous Federal Governments, prior to the current Government, there has been no commitment to realization of “Treaty and Voice” (Parliament of Australia, 2017). Unlike its settler colonial sisters within the former British Empire (e.g., Canada, South Africa), Indigenous Australians receive no mention as Australia’s First people within the Australian Constitution (1901) and the mythology of “terra nullius” remains enshrined in constitutional law.

Recent changes in government administration have resulted in the framing of a national referendum for constitutional reform for an Indigenous Voice to Parliament, which occurred in October 2023 (Fleck, 2023; Voice to Parliament, 2023). With the vote being unsuccessful, it is unknown at the time of publication how the resounding rejection of the Indigenous Voice to Parliament by the settler population will impact issues such as the severe rates of incarceration experienced by all Indigenous people, let alone the population focus of this research, First Nations Deaf people. Taking this as the research backdrop, our aim of this article is to illustrate the core concerns of First Nations Deaf advocates, activists, and professionals, working alongside as allies, in their attempts at intervening in the prison pipeline of extreme incarceration for First Nations Deaf that they represent.

## Research questions

To address these core areas of need related to First Nations Deaf in Australia and the criminal justice system, we asked three core research questions:

1. How have contemporary First Nations Deaf people historically accessed supports in the criminal justice system in Australia?
2. What is the role of communication needs of First Nations Deaf people in the Australian criminal justice system?
3. Assuming supports in the system are not as accessible as it should be under the law, what changes related to laws, policies, or practices might be necessary to render supports in the system more accessible to First Nations Deaf people?

Based on an initial round of semi-structured qualitative interviews, in this article, we illustrate core areas of need to address the ongoing injustices experienced by First Nations Deaf people.^3^ We highlight areas for redress related to: (a) indefinite detention and unfit to plead, (b) a need for an intersectional approach to justice, (c) applying the maximum extent of the law while minimizing social services-related resources, and (d) the need for language access and qualified sign language interpreters.

## Theoretical frameworks

For this project, to address the complex cultural and linguistic realities in Aboriginal communities, we utilized an interdisciplinary theoretical framework to inform our research methods. These main frameworks were: (a) Deaf Studies, (b) Critical Deaf Studies, and (c) Indigenous Disability Studies. Additionally, decolonizing methodology and community-based participatory research (CBPR) frameworks informed us in this work. Below, we describe how these frameworks, when taken together, have guided this project from conceptualization through to data collection, analysis, and presentation including coauthorship.

## Deaf Studies

Deaf Studies promotes the idea that the Deaf communities are a linguistic minority whose members share important cultural and historical traditions (Lane, 1995). The thread that unites this minority is the use of signed languages (e.g., Australian Sign Language [Auslan], American Sign Language [ASL]), which are standardized languages that Deaf people from all walks of life around the world use to communicate with each other (Henderson & Hendershott, 1991). A core principle of Deaf Studies centers on the idea that deafness is not a disability but a human condition that becomes a disability when the person intersects with a private or public policy or practice that fails to accommodate the person’s condition (Liasidou, 2013). These types of structural marginalization can be amplified when considering Deaf communities in the global South as evidenced by their experiences of linguistic oppression through the devaluing of Indigenous sign languages (McEwan, 2020).

## Critical Deaf Studies

Deaf Studies, traditionally as a field, has reflected a White, heterosexual, male, Northern perspective (Bauman, 2008). *Critical* Deaf Studies scholars, however, promote decolonizing practices and disability justice for Deaf people around the world and advocate for a reevaluation of explanatory paradigms; new terms of engagement in the struggle for social justice; and exploration of the role of positionality, power, and privilege (Bauman, 2008). Whether traditional or critical, Deaf Studies scholars ground their work on universal human rights that helps promote a fuller understanding of the core principles and concepts underlying disability rights, including Deaf rights. From the UN’s Universal Declaration of Human Rights (1948) to the Convention on the Rights of Persons with Disabilities (2006), the central idea is that human rights are universal (i.e., applying equally and without discrimination to every single human being on the planet); inherent (i.e., central to who you are as a human being); and, inalienable (i.e., automatically belonging to every single human being independent of a government’s judgment) (Lord, 2007).

Applying a Critical Deaf Studies framework in research is one way to closely examine the roles and relationships under White settler colonialism (Grech & Soldatić, 2015). The distribution and concentration of power that locks in poverty and marginalization for First Nations communities is a potential area for future research. Dr. Scott Avery, a Deaf Worimi man, suggests that research production on behalf of community advocacy must be centered on the concept of “narrative” storytelling as the message. To Avery (2018), “Narrative inquiry is a recognized approach within disability research through which the voices of people with disability are made central in shaping an understanding of the issues that affect them individually and socially” (p. 26). First Nations people with disability narrative combines three elements: (a) testimony,^4^ (b) statistical data,^5^ and (c) yarning^6^ (Avery, 2018, p. 26).

## Indigenous Disability Studies and Indigenous data sovereignty

This study was led by the conceptual work of Indigenous disability Australian scholars, including Gilroy and Donelly (2016), Esgin et al. (2019), Avery (2020), and settlers Fitts and Soldatić in collaboration with Indigenous colleagues, Yasmin Johnson, June Riemer, Jennifer Cullen, and Elaine Wills (2023a; 2023b). Indigenous Disability Studies scholars promote Indigenous ways of knowing, doing, and being, including Indigenous gendered practices of knowledge creation, in all phases of the conduct of research (see Martin & Mirraboopa, 2003). According to Gilroy and Donelly (2016), Indigenous Disability Studies scholars combine practices of respect and reciprocity with practices of Indigenous data sovereignty, recognizing that Indigenous stories and storying, both individually and collectively, always remain under Indigenous custodianship.

Through these approaches, Grech and Soldatić (2014) suggest that Southern disability theory moves beyond the Northern framing of disability studies through which “minimal attention [is] paid to cultures, context, and histories, and rarely responsive or even acknowledging Southern voices, perspectives and theories that have been developing as a counter discourse” (p. 1). Indigenous Disability Studies and data sovereignty practices are grounded in Indigenous strategies of engaging in narrative practices of engaging within, across, and through the world, bringing forth Indigenous strategies of justice, rights, and reparations in all aspects of the knowledge production process. Examples of Indigenous data sovereignty include ensuring that Indigenous people have the agency to make decisions related to how they govern data about them and that they control how they are represented in that data. This data must also be accessible to and controlled by Indigenous communities (Global Indigenous Digital Data Alliance, 2023).

Accordingly, in this article, we recognized the core role of First Nations Deaf knowledges to the production of the research itself, particularly through contributions of one of the coauthors of this article, Jody Barney, a First Nations Deaf woman, working at the intersections of the Australian criminal justice system to uphold the rights of First Nations Deaf people through actively facilitating Indigenous consultation and cultural interpretation. Rather than merely adjusting conventional disability studies practices ‘coproduction’ in the research process, Indigenous Disability Studies research is centered upon Indigenous systems of doing and being in the world, as they navigate settler legacies of power, violence, and mass injustice through the ongoing incarceration of Indigenous people in settler colonial Australia. Such research approaches recognize the history of settler colonial violence and the role of racialized science that engaged in white racist knowledge making practices that have legitimized the ongoing violence against Indigenous people of their lands, culture, and family (see Soldatić & Gilroy, 2018).

Given the foregrounding of Indigenous knowledge practices as central to the research process, next, we move to identify our own positionalities to enable readers to understand the potential impact on the research. We, as outlined below, consist of a diverse team, including Indigenous, nonindigenous, disabled, nondisabled, and Deaf community members. We also have different degrees of experience in the research domain. We seek to ground our research in understanding and amplifying some of the core issues facing First Nations Deaf people as they navigate attaining support within the criminal justice system, and to generate knowledge that Indigenous people can utilize for their advocacy locally, nationally, and within international forums.

## Decolonizing methodology and CBPR

As a part of the overall theoretical framework of the project, it is important to note that we view decolonizing methodologies and CBPR approaches to inquiry as not only a *method* for this work, but also useful *theoretical lenses* through which to view social justice approaches to the realization of disability rights. In particular, our work is rooted in decolonizing methods and CBPR as useful approaches in thinking about how to engage Indigenous and non-Indigenous stakeholders in the system of justice reform process.^7^

To address the settler colonial realities of Australia, we drew on decolonial Indigenous disability standpoint theories as outlined above as well as critical cultural positionalities of postcolonial theories in recognition that formerly colonized people cannot return to their precolonial ways of being (Fanon, 1963; Hall, 1990) and therefore, live, breathe, and work at a cultural interface that navigates Indigenous knowledge practices and ways of being in the world with settler colonial regimes of power (see Nakata, 2002^8^). Indigenous scholar Nakata (2002) poses his theory of the “cultural interface,” through which he attempts to move beyond Fanon’s (1963) postcolonial position that articulates “individuals without an anchor” who cannot return to their precolonial roots (p. 176). While this has relevance for Indigenous people’s within settler colonial contexts, Nakata’s (2002) cultural interface seeks to acknowledge the critically important ways in which Indigenous people within settler colonial societies have maintained their ongoing engagement with their own knowledge systems, despite the violence of settler colonialities that have been specifically designed to eliminate the “native,” that is, replace Indigenous people’s species and lands with European settlers, plants, and life (Wolfe, 2006). Working at the cultural interface this project incorporated a range of First Nations Deaf cultural ways of engaging, being, and doing knowledge making (see Avery, 2018) including: (a) conducting research in the local language (Skutnabb-Kangas, 2000), (b) promoting local ways of knowing, and (c) encouraging Indigenous participants to direct the research (Smith, 1999).

Decolonizing methodologies involve a range of approaches that are rooted in CBPR collaborative research techniques. The Indigenous research participants in this project emphasize community collaboration and that such collaborative practices are maintained throughout not just to reflect the critical role in Indigenous knowledges and experiences are generative of new knowledge frameworks but also to ensure that Indigenous sovereignty of the research is maintained throughout each stage of the work. The goal is to create solutions with clear and immediate application to local communities (Israel et al., 1998; Stanton, 2014).

## Positionality

### Brent Elder

Brent’s positionality is inherently tied to Western understandings of disability and deafness. As a result, he understands that acknowledging these realities is important. Because of the privileges he benefits from as a White, nondisabled, academic, from a country with a strong history of colonization, he understands that his role is not to represent nor speak for colonized people. However, he strongly believes in leveraging these privileges to his partners in the global South through transnational collaboration in ways so that historically marginalized groups have allies committed to decolonizing practices and disability justice outside of their respective communities (Kincheloe & Steinberg, 2008). Through international decolonizing research projects, he tries to be actively aware of how his work may perpetuate marginalizing and neocolonial systems. While his outsider status in research is unavoidable due to his experiences in the Northern academy, he does have extensive experience conducting transnational decolonizing research and CBPR projects around the world.

### Karen Soldatić

Karen is first-generation settler Australian and is a descendant from the Istro-Romanian peoples, a minority group from the Istrian region of what is now known as Croatia. Karen’s engagement with Indigenous disability incarceration is thus shaped by the lived experience of intergenerational socialization within Australia and the disabling impacts of settler colonial practices in regard to the racialization of immigrant children under the White Australia policy in the early years (see Parliament of Australia, 2009).

### Michael Schwartz

Michael is a Deaf law professor who is director and supervising attorney of a disability law clinic at Syracuse University College of Law, and who has been deaf since birth. He brings to the project his lived experiences with discrimination based on disability and identifies himself as a native insider intimately connected with and part of the international Deaf community. Michael is fluent in ASL and conversational in British Sign Language. As a Deaf person, he is a walking magnet for discriminatory behavior of people with typical hearing who either advertently or inadvertently fail to respect his autonomy and freedom. Acutely aware of neocolonial and marginalizing systems of power, he is cognizant of his privilege and power as a well-educated White male who has social, political, and economical capital in reserve for his struggles against systems designed by those who are not deaf. Michael has experience with transnational decolonizing CBPR research in the UK, Australia, and the United States.

### Jody Barney

Jody is a proud Birri-Gubba/Urangan and South Sea Islander Deaf woman. Globally recognized for her work in the First Nations Deaf space across Justice, women’s rights and fluent in 20 Aboriginal Sign Language systems in Australia. She is an Atlantic Fellow of Social Equity and a current Churchill Fellow researching on the impact of First Nations Deaf people’s access rights to cultural sign languages in custody. As a well-respected Aboriginal consultant in Australia, she’s had access to over 200 communities for the last 35 years. Her connections, lore (law), and customs allow her intimate knowledge of those who are directly impacted by these barriers.

### Damien Howard

Damien is a non-Indigenous professional who grew up in a mainstream large city environment steeped in the racist narrative about Indigenous people where he had never met an Indigenous person until communing the Northern Territory 40 years ago. Over that 40 years, he has worked with First Nations colleagues who have generously mentored him to better understand First Nations cultural perspectives. For the last 15 years, he has worked with Jody Barney as part of a bicultural team working to achieve solutions, often for First Nations Deaf people within the criminal justice system.

### Patrick McGee

Patrick is a non-Indigenous disability and justice systems advocate working with First Nations Australians with disability who are involved in the criminal justice system in the Northern Territory. Patrick is particularly interested in how First Nations cultural safety and cultural protocols can support people with disability who may be a risk of harm to others. Patrick was recently awarded a Churchill Fellowship to investigate how to dismantle and replace indefinite detention in Australia (see McGee et al., 2024).

## Methods

In the following sections, we describe our methodology for the project. Our approaches were informed by the frameworks noted above (e.g., Avery, 2018; Fitts et al., 2023, 2023b; Gilroy et al., 2018; Gilroy & Donelly, 2016; Grech & Soldatić, 2015; Nakata, 2002; Smith, 1999; Stanton, 2014).

### Sites of study

This initial iteration of the project took place in Sydney in May 2023. While Elder, Soldatić, and Schwartz conducted some qualitative interviews in-person, they also conducted some interviews virtually from various locations throughout Australia. They plan to include the data from those interviews in subsequent publications. While the focus of inquiry of this initial iteration of this ongoing project was largely focused on *organizations* that engage in various aspects of justice with Aboriginal communities around Australia, as trust is built within these communities, our goals are to more deeply engage with First Nations Deaf people to align more closely with Indigenous CBPR methodologies that maintain Indigenous data sovereignty goals.

### Data collection and participants

As noted above, while the goal of the project is to eventually engage with individual members of First Nations Deaf communities throughout Australia, to build trust within these communities (Avery, 2018), as well as momentum within this project, Elder, Soldatić, and Schwartz began interviews with organizations and individuals engaged in various aspects of justice work with Aboriginal communities. Specifically, Soldatić and her colleagues provided contacts for participants through their respective contacts working in related areas of justice for Aboriginal communities. Some participants were active members of Aboriginal justice organizations, while others engaged in interviews while reflecting on past experiences working as allies in collaboration with members of Aboriginal communities. Notably, Jody Barney joined as a coauthor on the article after an informal phone call where she described her deep experiences with First Nations Deaf communities, as well as her identity as a First Nations Deaf scholar and activist. Additionally, two of the participants (i.e., Damien Howard and Patrick McGee) evolved into coauthors as their expertise and value they added to the project warranted authorship. This flexibility in authorship is important to highlight as coproduction of knowledge and coauthorship are also critical components of CBPR (Elder & Odoyo, 2018; Stanton, 2014). While Elder, Soldatić, and Schwartz did not interview members of the First Nations Deaf communities, all participants were experts/allies in the disability/Aboriginal justice sector. See Table 1 for a description of participants and their relation to Aboriginal disability justice.

**Table 1:** Participant description and experience collaborating with Aboriginal communities

**Name**	**Member of aboriginal community**	**Disability/Aboriginal** **justice sector**
Participant 1	N	Advocate for First Nations people with disability
Participant 2 (Patrick McGee^a^)	N	Churchill Fellow, National Manager Policy Research Advocacy at Australian Federation of Disability Organizations
Participant 3 (Damien Howard^a^)	N	Phoenix Consulting, ally of First Nations people with disability
Participant 4	N	Advocate for Aboriginal community health
Participant 5	N	Human rights lawyer
Participant 6	N	Disability rights lawyer

^a^This participant is also a coauthor.

### Interview procedures

Elder, Soldatić, and Schwartz collectively drafted and edited semistructured qualitative interview questions. They crafted questions around the research questions that focused on First Nations Deaf peoples’ experiences interacting with the criminal justice system. Then, they included interview directions at the top of the questions to ensure a common approach to asking questions to participants. See Appendix A for a sample of the interview question protocol. While there were not any Deaf participants, Michael Schwartz is Deaf, which meant two ASL interpreters were a part of the research team and interview process.

Elder, Soldatić, and Schwartz’s general interview protocol was to start by asking consistent open-ended questions that led to narrower, more focused questions based on initial participant responses. Schwartz led most of the interviews during which he would sign the question in ASL, and the ASL interpreter would voice the questions to participants. This communication chain was reversed when the participant responded. The second ASL interpreter would support the accuracy of the interpreting process by adding clarifications as required during the interviews. For transcription purposes, Elder recorded the interviews on his phone and took written notes and would occasionally ask clarifying questions along with Soldatić. When Elder or Soldatić led interviews, the interpreting process remained, and Schwartz would ask clarifying or extending questions. Following each interview, Elder, Soldatić, and Schwartz, along with the ASL interpreters, would craft memos to ensure the team was consistently making meaning from the participants’ stories.

### Data analysis

During the 3 weeks of data collection, Elder, Soldatić, and Schwartz held discussions on emerging themes after reading interview transcriptions. These discussions established a framework for open coding. Elder, Soldatić, and Schwartz used a constructivist grounded theory approach and constant comparison method (Charmaz & Mitchell, 2001) to analyze data. This allowed them to simultaneously collect and analyze data, with a focus on how participants construct meaning in relation to the area of inquiry (Charmaz, 2005; Chun Tie et al., 2019). The Elder, Soldatić, and Schwartz then generated more abstract concepts and theories about the emerging data through inductive processes (i.e., coding). (Charmaz, 2005). In this case, Elder, Soldatić, and Schwartz used the data to inform a plan of action to inform the next steps in their activist-related agenda as well as the next steps in their research agenda. Elder, Soldatić, and Schwartz used coding methods as outlined by Bogdan and Biklen (2007) and coded data in three phases: (a) open coding, (b) axial coding, and (c) selective coding, which helped them identify four significant themes in the transcripts (Creswell, 2013). It is important to note that Barney, Howard, and McGee did not participate in the data analysis as they joined the project after initial data analysis. However, all coauthors provided feedback on the emerging themes and provided feedback on interpretation of participant excerpts that helped them collectively interpret and make meaning from the data.

### Open coding

Elder, Soldatić, and Schwartz read each interview transcript and applied open codes to the topics participants discussed. After comparing notes on each interview, Elder, Soldatić, and Schwartz discussed the emerging thematic concepts discussed by the participants. Sample open codes they commonly applied to interviews included: (a) a government denial of Aboriginal people civil and human rights, (b) settler colonial violence, (c) overlapping systems of oppression (e.g., poverty and racism), and (d) a lack of qualified Auslan and Aboriginal sign language interpreters.

### Axial coding

During the axial coding phase of the analysis process, Elder, Soldatić, and Schwartz identified participant excerpts that spoke more powerfully to the research questions and aims of the project. They read through the excerpts, and they narrowed down the quotes that most cohesively centered the perspectives of the participants related to Aboriginal access to supports in the justice system. Then, Elder, Soldatić, and Schwartz identified the top four codes they applied to participant excerpts. They identified these as themes and included: (a) indefinite detention and unfit to plead, (b) a need for an intersectional approach to justice, (c) applying the maximum extent of the law while minimizing social services-related resources, and (d) the need for language access and qualified sign language interpreters. In alignment with Indigenous data sovereignty practices, Elder, Soldatić, and Schwartz held an Aboriginal Disability Community Forum to share emerging data and to gather feedback from members of Aboriginal communities. During this forum, members of various Aboriginal groups shared their community’s personal experiences with discrimination and marginalization which aligned with the emerging data (see Elder et al., 2023a).

### Selective coding

At the beginning of the selective coding process, Elder, Soldatić, and Schwartz read each excerpt and collectively identified the top five excerpts for each emerging theme, which were later affirmed by conducting member checks (Lincoln et al., 1985) with participants who contributed the respective quote. This process allowed Elder, Soldatić, and Schwartz to identify up to five excerpts per theme that most powerfully illuminated each finding. Elder, Soldatić, and Schwartz chose excerpts for each finding that most accurately represented the participants’ stories.

## Findings and discussion

In this section, we present findings in the following four key themes: (a) indefinite detention and unfit to plead, (b) a need for an intersectional approach to justice, (c) applying the maximum extent of the law while minimizing social services–related resources, and (d) the need for language access and qualified sign language interpreters. In the following sections, we introduce each theme, provide participant quotes in support of their findings, and make connections to relevant literature.

### Theme 1: indefinite detention and unfit to plead

For those in the Aboriginal community experiencing the Australian system of justice at the intersection of deafness and disability, they face particularly precarious consequences for being a disabled person of color. Those who are labeled as “cognitively impaired” or “mentally ill” risk being “unconvicted,” which can result in indefinite detention and under maximum security in prison.^9^ The Australian justice system couples the punishment with a disabled person’s fitness, or lack thereof, to plead to the criminal charges against them (McGee et al., 2024). When asked about indefinite detention, Participant 5 said,

[Australia’s criminal justice system is] a pretty unfavorable regime. If you are found to be unfit, then you can be subject to indefinite detention or community supervision orders which can be quite oppressive. For a lot of people, particularly if the evidence against them is very strong, they may be better to plead guilty, do their time and be done with it rather than be subject to constant supervision which can see them in and out of custody for long periods of time, and longer than they would otherwise be under sentence.

Here, the participant is saying that it is better for a criminal defendant to plead and go to trial than to be adjudicated “unfit to plead” and spend years in custody without trial and a verdict of guilty. Rather than being a disability-related indefinite sentence, according to the Australian Human Rights Commission (2016), the outcome of an “unfit to plead” should depend on the circumstances and evidence against the defendant rather than the presence of a perceived disability or the court’s inability to appropriately accommodate the Deaf and/or disabled defendant. Consider this quote by Participant 2,

If it is a mental illness [it is] in the forensic mental health process system. [If] it is a cognitive disability [it is] in the disability forensic system...It does not matter if you shoplift or if you kill someone, you will end up indefinitely detained. The period of detention is out of proportion with the crime committed. So, you can find people in our forensic detention systems that have shoplifted and are there seven years later.

This participant’s quote aligns with findings in a report on the rights of Indigenous people by the Human Rights Council at the UN (Office of the High Commissioner on Human Rights [OHCHR], 2019). According to First Peoples Disability Network Australia (FPDN) (2021), “Aboriginal and Torres Strait Islander people with disability are 14 times more likely to be imprisoned with one third reporting a disability, 50% reporting a history of psychosocial disability, and 25-30% of prisoners having an intellectual disability” (p. 1). It is important to note that these statistics do not suggest that 75%–80% of incarcerated First Nations people have disabilities; rather, they suggest many report disabilities, and some of those people may be reporting that they experience more than one type of disability at once (i.e., comorbidity). As noted by this participant, these statistics highlight a problem with these forensic systems.

During interviews, participants repeatedly stated that a barrier in Australia’s system of justice is that the enforcement of the UNCRPD (2006) is underutilized. When asked about how international legal mandates like the UNCPRD can be better leveraged to promote disabled Aboriginal justice, Participant 6 said,

Complaints that have been made against Australia to the [UNCRPD] committee with respect to the participation within the criminal justice system have predominantly concerned indefinite detention. In particular, two complaints have been brought to the [UNCRPD] committee around the indefinite detention of people with cognitive disability which is a significant legislative issue facing First Nations people. Both of those persons were First Nations people but neither of them was deaf.

While complaints have been filed against how the Australia criminal justice system has indefinitely detained Aboriginal people with intellectual disabilities, this has not been challenged for indefinitely detained First Nations Deaf people. As underscored by the Royal Commission Report on Disability (2023), under the UNCRPD (2006), Australia is obligated to protect people with disability, including Indigenous people within the criminal justice system. This report (2023) affirms that “Article 14(2) of the UNCRPD requires States Parties to ensure, where people with disability are deprived of their liberty, they are to be treated in compliance with the objectives and principles of the CRPD” (p. 5).

### Theme 2: a need for an intersectional approach to justice

A common refrain from the participants during interviews was that the barriers related to First Nations Deaf access to justice intersected and overlapped with other marginalizing systems of oppression in Australia. As Participant 1, a prominent advocate for First Nations people with disability, stressed, advocacy for the rights of Aboriginal people with disability requires an intersectional approach. This participant notes, through this perspective, the lens of Indigenous rights must include a sensitivity and understanding of what navigating the system is like for people with disability who struggle with access to supports and services and inclusion,

You might have a community-controlled legal service, but they themselves do not have a strong understanding of the experiences of First Nations people with deafness or with other disabilities. So, there will be very good understanding [of] the Indigenous rights, but they will not have a strong understanding of the disability rights and access and inclusion and the experiences of the First Nations person who is also deaf.

As Avery (2018) points out in his book, *Culture is Inclusion*, “intersectional inequality is acute and pervasive across all supports for Aboriginal and Torres Strait Islander people with disability; including disability services, health, education, employment, housing, and transport” (p. 108).

When asked about potential pathways to the criminal justice system for First Nations Deaf people, Participant 3, a long-time advocate and ally of the disabled Aboriginal community, said,

The first problem is over access to the system in that Deaf First Nations people who have not had well-developed communication systems without education are liable to come to the attention of the criminal justice system because they have multiple reasons. They are scapegoated by their family to be set up to take the fall for the other’s criminal behavior. They are unable to get their wants met so that they are more likely to steal or be coercive and be physically involved in fights. That brings the attention of the criminal justice system.

Here, Participant 3 identified a complex set of barriers impacting First Nations Deaf people and their interactions with the criminal justice system including: (a) lack of education, (b) abuse by family members, and (c) engaging in criminal behavior to get their needs met. Such preexisting socioeconomic factors can pave the pathway to jail, which can be amplified by the system’s failure to provide qualified, culturally competent Aboriginal sign language interpreters skilled in the person’s sign language and cultural milieu (Power, 2013; Vanderpoll & Howard, 2012).

Related to barriers to education, according to Participant 3,

The research does show that low education levels are a pathway to prison…94% of indigenous First Nations inmates had hearing loss.^10^ And mostly they were not aware of it, and no one else in the prison was aware of it.

This quote is supported by literature that identifies low First Nations education levels as a pathway to prison in Australia (O’Brien & Trudgett, 2020) and is connected to a more global issue of disproportionate incarceration of Indigenous people, which is not confined to Australia (Austin et al., 2020). Additionally, literature suggests that First Nations people view Western hearing assessments as culturally unsafe,^11^ in addition to the reality that hearing-related disabilities is so common in First Nations families that it has been normalized and not viewed as something to be addressed, which is one potential contributing factor as to why First Nations inmates and prison workers are unaware of this issue (Royal Commission Report on Disability, 2023).

Gaining effective access to education and bilingual education, whether Deaf, disabled, or not, is critical for children whose primary language(s) is not English (Rahman, 2020). In the next quote, Participant 4 describes the educational and linguistic context for many Aboriginal children in Australia.

Children in remote communities often speak several Aboriginal languages. They may learn a bit of English before they go to school but then schooling is in English. There is Aboriginal education assistance in the classroom to help bridge that gap, but it is not usually bilingual education. For Aboriginal people living in urban areas or in regional areas where often historically they were denied access to their language by policies that were in place at the time. Aboriginal English is what they speak, which is a language in its own right.

The lack of access to appropriate education puts Aboriginal children at a disadvantage (Deafness Forum Australia, 2022). These disadvantages include low school attendance, decreased school readiness, and overall lower academic achievement (He et al., 2019; Su et al., 2020). These realities underscore the need to promote educational programs that are culturally responsive to First Nations families so there is decreased stigma related to supporting the disability-related needs of their children (Deafness Forum Australia, 2022).

### Theme 3: applying the maximum force of the law while minimizing social services–related resources

In addition to the complex, intersectional nature of First Nations Deaf oppression, participants routinely discussed that when disabled Aboriginal people had interactions with the system of justice, they experienced the maximum force of the law. Calling attention to this alarming and punitive reality, Participant 2 said, “30% of any detained population in this country are First Nations Australians. Despite being 1% or 2% of the population, their incarceration rate is a third of the population of people detained.” This quote is borne out by statistics showing that the Aboriginal and Torres Strait Islander communities accounted for 3.8% of the total population of Australia but constitute 32% of all prisoners (ABS, 2021). And, according to the Australian Human Rights Commission (2021), “Indigenous people were 17.3 times more likely to be arrested than non-Indigenous people. The over-representation rate in Western Australia is four times the national average” (p. 1).

Participant 2 had this to say about this disproportional representation of First Nations people in the prison system,

We are using the maximum amount of legislative authority, the maximum amount of legislative power, the maximum amount of force to detain a group of people who in fact probably have never received the right amount of support in the first place, enabling them to make different choices from the ones that have led them into being detained in the way that they have been detained. And so, you know, lowering the amount of effort that we make or that we take into detaining people in these ways and upping the level of the support that we provide them is really their way out of this quagmire that we have created for ourselves.

As noted by this participant, lowering force and increasing access to social services resources for Aboriginal communities offer one way forward in pushing back against this noted disproportionality (Jones et al., 2023).

To decrease the maximum force of the law and increase social services for Aboriginal people with disability, Participant 1 described the complexities of what those realities can involve by stating,

The funding is at a Commonwealth level and it is through different departments. So, you will have funding that comes from one department to do with health, one department to do with early childhood, one department to do with education. Then you have the states and territories who also fund different aspects of that. So, the funding is really complex, messy, and siloed.

Funding systems that are already “complex, messy, and siloed” means that the existing systems through which state and territories fund disability through their criminal justice systems, including corrective services, juvenile justice, and justice health agencies, are already challenging to navigate. That impacts negatively on people with disabilities who require these services. According to the Royal Commission Report on Disability (2023), the intersections of funding between the National Disability Insurance Scheme and the criminal system “contributes to uncertainty and confusion” about funding (pg. 11).

### Theme 4: the need for language access and qualified sign language interpreters

One way to increase access to communication supports in the justice system and decrease the human rights abuses experienced by the First Nations Deaf community is to promote government schemes that train people to be qualified Aboriginal sign language interpreters (Deafness Forum Australia, 2022). This potential way forward could provide First Nations Deaf people with more effective communication systems as they interact with the system of justice. The dearth of qualified Aboriginal sign language interpreters in Australia is described by Participant 2,

In this country, we have got Mandarin interpreters. We have got Arabic interpreters. You can get Tagalog interpreters. But you cannot get Aboriginal interpreters with expertise to interpret. Finding Auslan interpreters who can provide [sign] language access for Aboriginal people is very difficult.

To further underscore the lack of qualified Aboriginal sign language interpreters, when asked if there are any existing training programs on Aboriginal sign languages, Participant 3 stated, “Absolutely none.” This quote underscores the need to ensure that Auslan interpreters have cultural sign language training (Deafness Forum Australia, 2022; Power, 2013). While there is a lack of interpreters in general, there are none who are completely qualified (i.e., completed a First Nations Deaf interpreter program) to work *unaided* by a First Nations Deaf interpreter, such as Jody Barney and the other two First Nations Deaf interpreters in the country (Disability Royal Commission, 2019).

The theme of this crucial gap in training is repeated in the following quote by Participant 6 when they say, “No is the short answer. There is a significant gap between having judges and lawyers trained in knowing and importantly implementing the CRPD into trial settings within the criminal justice system. There is a significant gap.” This quote, a response to the question about CRPD training, frames the need for training for judges and lawyers on how to apply the CRPD in the system of justice (Brolan & Harley, 2018).

In addition to the need for Aboriginal sign language interpreters, Participant 3 elaborates on the need for early language support for First Nations Deaf children,

Often with Deaf First Nations people the problem is that no one has established the communication support that the person needs so by the time they grow up old enough to be in front of the court system no one in that system can communicate with them.

A lack of an effective communication system has significant ramifications when it comes to interactions with the justice system as judges and lawyers do not have the skills required to support First Nations Deaf access to communication in court proceedings (He et al., 2019). Consider this quote by Participant 5,

One of the problems, from a criminal justice perspective, is that even if there is an interpreter available for a client and the interpreter can effectively interpret at the level required for criminal proceedings, it is still possible that the judge, the prosecutor and even the defense, may themselves lack the cross-cultural literacy required to ensure that a person gets a fair hearing and a just outcome.

This quote echoes the barriers to justice outlined in the Deafness Forum Australia (2022) when it states,

Police, court staff and prison staff often mistake hearing loss for a mental health issue and have little or no knowledge of the signs of hearing loss or the high prevalence of hearing loss in the Aboriginal and Torres Strait Islander population. (p. 10)

These realities point to the need not only for more First Nations Deaf interpreter programs but also training for justice-related stakeholders on reasonable adjustments within court proceedings (Deafness Forum Australia, 2022; Royal Commission Report on Disability, 2023).

### Implications

To guide this section, we have organized the implications around each theme in relation to how participants responded to implications-related interview questions (see questions #9 and #10 in Appendix A). In the following sections, we present the implications of this work based on participant perspectives of what could and should be the next steps based on their expertise working on these issues in partnership with First Nations Deaf communities.

### Indefinite detention and unfit to plead: adopting international models from similar contexts

Some participants suggested that in order for the Australian government to separate the notions of fitness to plead and indefinite detention, Australia should look to Canada to see what they have done to decouple this policy. In Canada, courts apply the Gladue Principles (Government of Canada, 2023), which means that when a First Nations Canadian appears in a Canadian court, the judge is required to take into account the impact of colonization on the circumstances that brought that person to court as well as to consider any cultural alternatives to incarceration. The implications of these considerations is that the Gladue Principles (Government of Canada, 2023) require acknowledgment of the role of colonialism in shaping a defendant’s behavior. Given Australia’s past, examining the impact of colonialism on the Aboriginal communities could be a significant step forward. Interrogating and challenging the coupled policy of “unfit to plead” and “indefinite detention” offers an opportunity to create a system of justice in Australia that is more equal, equitable, and inclusive (McGee et al., 2024).

### Intersectionality: understanding and identifying the complexity of indigeneity, disability/deafness, and the criminal justice system

For stakeholders in the criminal justice system to better understand the intersectional and oppressive systems at play when considering First Nations Deaf access to justice, some participants emphasized the need to build capacity for those in the legal system (e.g., police officers, judges, lawyers, prison officials) about the intersections of race and disability (Brolan & Harley, 2018). Participants specifically mentioned Disability Studies Critical Race Studies (DisCrit) as one potential guiding lens (Annamma et al., 2013) for such professional development. One way to realistically do this is to require such topics in the professional development provided in judicial conferences around Australia. The implications of this is that judges and other justice stakeholders could participate in ongoing discussions and professional development on Aboriginal disability justice, *and* these recurring professional learning sessions should be developed and led by Deaf and disabled Aboriginal leaders (see Jones et al., 2023).

### Public policy investment: applying the maximum force of the law while minimizing social services–related resources

As noted previously by the participants, *decreasing* the force of the law as applied to First Nations Deaf communities and *increasing* their access to culturally appropriate social services could have significant implications over the long term. Divesting in punitive models of criminal justice and diverting public resources to social service pathways can be one effective way to bring down the disproportionate incarceration rates for Deaf and disabled Aboriginal people (Deafness Forum Australia, 2022). Some participants shared their firsthand experiences of witnessing the implications of providing incarcerated First Nations Deaf appropriate social support and language access rather than focus on punitive policing and rapid criminalization of non-normative behaviors.

As noted by one participant, the overall implications of this approach can mean that First Nations Deaf people can then be provided with increased access to services and supports that are socioculturally familiar with appropriate language supports, including nonverbal communicative techniques such as cultural language and gestures and facial nuances better able to acquire reliable and comprehensible language (Howard & Hampton, 2006). Such language skills have strong implications well beyond simply navigating the system of justice.

### The need for language access and qualified sign language interpreters

When reflecting on the implications of providing training for stakeholders in the system of justice, some participants suggested practical ways in which more qualified interpreters could be certified in Aboriginal signed languages and Auslan. Some practical ways forward would be to: (a) build communication portfolios around First Nations Deaf people to capture their language, systems of communication (including home signs, gestures, and Auslan) and (b) leverage the expertise of community-approved First Nations Deaf sign language users. This means that professionals in a variety of fields (e.g., psychologists) could partner with people in First Nations Deaf communities, learn about the specific needs of these communities based on their cultural and linguistic priorities, and collaborate to center the needs of these communities while still providing critical social services. Not only could this redistribute power and agency to First Nations Deaf communities, but it could also build partnerships between Deaf and hearing professionals in ways that could inform and impact other facets of Indigenous life beyond the justice system.

## Conclusion and next steps

As Elder, Soldatić, and Schwartz concluded their interviews, they asked participants what they felt the next steps of this work should be. Participants unanimously suggested that amplifying these stories on the international stage was one way to pressure the Australian Government to act. To do so, in this section, we describe steps the Australian government could take to better realize the rights of First Nations Deaf people throughout Australia.

During interviews, some participants mentioned when the UN Subcommittee for the Prevention of Torture visited Australia in 2022 to investigate torture of people in detention, including Aboriginal people with disability who were indefinitely detained, subcommittee members were prevented from accessing detention centers and subsequently canceled their formal visit. With the visit canceled, this called into question the usefulness of the supposedly robust mechanisms with the UN to advocate for people who are illegally detained. This denial of a formal visit also further diminishes the transparency and accountability of the system of justice in Australia, further perpetuating the notion that it is really a closed system rife with cruel, degrading, inhumane treatment of Australia’s most vulnerable citizens.

If an organization like the UN cannot enforce the CRPD, what does that mean about the ability of local community advocates and their allies to effectuate change? In addition to the citations we have provided throughout this article, these participants have offered additional evidence that highlights that Deaf and/or disabled First Nations people have a significantly increased risk of interacting with the Australian criminal justice system which can lead to cruel, degrading, and (indefinite) inhumane treatment (Deafness Forum Australia, 2022; Royal Commission Report on Disability, 2023). As a group of scholars committed the redistribution of power through Indigenous-led CBPR research, we believe that criminal justice systems like Australia’s must be challenged and brought to account for its abusive treatment of Aboriginal and Torres Strait Islander communities.

To amplify the work of the professionals who participated in this project, in addition to publishing this article, Elder, Soldatić, and Schwartz have coauthored two blogs as a way to get information about this ongoing project into First Nations communities and international communities as the articles go through the review process (see Elder et al., 2023a; Elder et al., 2023b). Additionally, we have applied for smaller-scale Australia-based grants that can intentionally bring leaders from the First Nations communities, including Deaf and disability communities, to focus on some of the pressing issues we present in the article. As a result of this ongoing project, Elder, Schwartz, and Barney copresented at a perceptions of deafness conference in Belfast in 2024, and Barney came to North America to visit Elder and Schwartz to discuss ways to promote this work to Indigenous communities in the United States, the United Nations, the World Bank, and the United States Agency for International Development (USAID). Finally, as we publish the varied body of work from this project, we intend to present it at the annual meeting of the Conference of States Parties (COSP) to the CRPD that takes place in New York every June. It is through these collective and sustained actions that we plan to begin bringing some of the implications of this work to fruition. As evidenced by the recent defeat of the Indigenous Voice to Parliament (2023), reform of the Australian system of justice as it pertains to Indigenous populations has a long way to go.

## Footnotes

1 We use the term, “First Nations Deaf” to refer to the Deaf members of the Aboriginal and Torres Strait Islander communities. Capitalizing the letter, “D,” as in “Deaf” connotes deafness as a cultural identity centered on fluency in sign language, whereas the small letter, “d,” as in “deaf” references hearing-related disability as a physical condition (Gregory & Hartley, 1990). We define the criminal justice system to include legal aid, police services, judicial processes, and prison programs. Additionally, to honor the variety of ways in which Aboriginal communities self-identify, we use terms like “Aboriginal,” “First Nations,” and “Indigenous” depending on how participants were using the terms and the context in which the terms were used by participants throughout the project.

2 The Optional Protocol is the mechanism through which individuals can file a complaint with the CRPD Committee and ask for an investigation on rights abuses.

3 While we discuss this later in the article, it is worth noting here that we did not interview any members of the First Nations Deaf community. While the goal of the project is to eventually engage with individual members of these communities throughout Australia, to build trust within these groups, we began interviews with organizations and individuals engaged in various aspects of justice work with Aboriginal communities.

4 Testimony: Testimony consists of first-hand oral testimony from Aboriginal and Torres Strait Islanders about their lived experiences with disability-based discrimination, and this includes stories told at forums and other gatherings. However, the invisibility of people who are deaf and hard of hearing can limit the “narratives” that emerge about hearing-related disabilities. This invisibility acts to restrict the narrative to people who are Deaf (i.e., those who use sign language or those who rely on lipreading). The narratives of this population are effectively largely silenced in First Nations communities. Without this narrative being available in research or advocacy, it cannot easily emerge within policy agendas (personal communication, 2023).

5 Statistical Data: Statistical data refer to government-gathered data like the Australian Bureau of Statistics (ABS), information that sheds light on the challenges facing Aboriginal and Torres Strait Islander people with disability. There are two other principal data sources relied on by Avery: (a) the National Aboriginal and Torres Strait Islander Social Survey (NATSISS) and (b) the Survey of Disability Ageing and Carers (SDAC) (Avery, 2018, p. 26).

6 Yarning: Yarning is an Aboriginal concept of storytelling whereby when asked, “What is your story?,” the speaker recounts their experiences at their own pace, on their own terms, and in a safe space (Avery, 2018, p. 11). It is critical for scholars and advocates in Deaf Studies, especially in white settler colonial context of Australia, to pay close attention on how to honor and respect the stories of Aboriginal and Torres Strait Islander communities as they deal with the intersections of racism, poverty, and ableism (Brolan & Harley, 2018).

7 Given that this was the first iteration of this project, it is important to note that Elder and Schwartz are outsiders to the First Nations Deaf communities, and the “community” that informed the CBPR aspects of this project were organizations and individuals who work in various Indigenous Deaf and disability spaces throughout Australia.

8 In this section, we intentionally cite foundational postcolonial scholars (e.g., Fanon, 1963; Hall, 1990) and Indigenous scholar Nakata (2002) to honor their contributions that have allowed space for this work to take place. Through our citation of these scholars, we do not wish to exclude other scholars that have contributed greatly to this field (see Barney, 2016a, 2016b; Cripps et al., 2010; Gilroy & Donelly, 2016; Gilroy et al., 2013; Gilroy et al., 2018; Howard & Barney, 2020; Jaffe & John, 2018; Puszka et al., 2022).

9 Often, non-Aboriginal professionals and their support systems did not have the capacity to access interpreters who could translate First Nations signs, so the accused could be effectively questioned. This inadequacy in having the needed communication supports frequently result in a judgment that the “the accused” is unfit (Howard, 2016; McGee et al., 2024; NAAJA, 2017).

10 From a study by Howard and Vanderpoll (2012) on inmates in Northern Territory correctional facilities, Howard found more than 90% of First Nations inmates had significant hearing loss.

11 See the Royal Commission Report (2023) entitled the *Wangkiny Yirra “Speaking Up” Project: First Nations women and children with disability and their experiences of family and domestic violence* for more information on the “Stolen Generation” and violence enacted upon First Nations women and children with disability.

## A. Appendix A

Interview Protocol

Begin with appreciations and ask about preferred Indigenous language terms to use (e.g., Aboriginal and Torres Strait Islanders, First Nations People). Then, discuss consent forms, 30–45-minute interviews, etc.

Questions:

1. Tell us a little about yourself and how you came to be connected to the First Nations Deaf community.
2. How do you refer, in terms of disability-sensitive terminology, to First Nations Deaf people? Person-first language?
3. How have contemporary First Nations Deaf people historically accessed the criminal justice system over the last 20–25 years?
4. What are some of the strategies First Nations Deaf people use to access the criminal justice system? Or adapt to the lack of access?
5. From your perspective, how do First Nations Deaf people understand their rights under the Disability Discrimination Act and the CRPD? Are there any gaps we should be aware of?
6. Do you think the services, programs, and activities of the criminal justice system are accessible to First Nations Deaf people? If not, where are the gaps?
7. Given a chance to meet police officers, prosecutors, defense council, judges, and prison officials, what would you tell them? What do you think First Nations Deaf people would tell them?
8. “Effective communication access.” What does that mean to you?
9. What changes/reforms to laws, policies, and practices would be needed to ensure effective communication access for First Nations Deaf people?
10. What are the next steps for ensuring more equitable access to the criminal justice system for First Nations Deaf people?
  - In what ways can communication access for First Nations Deaf people increase and be more effective?
  - How can there be greater financial resources allocated for training interpreters, justice officials, and First Nations Deaf people?
  - What specific criminal justice system reform is necessary so policies and practices greater compliance with the DDA and CRPD?
11. In what ways do you think our project can benefit the First Nations Deaf community?
12. Do you know of any disabled First Nations artists that might be interested in working with us to design an art piece to symbolize this project?
  Do you have any questions/comments for us?
